# *De Novo* Assembly of Complete Chloroplast Genomes from Non-model Species Based on a K-mer Frequency-Based Selection of Chloroplast Reads from Total DNA Sequences

**DOI:** 10.3389/fpls.2017.01271

**Published:** 2017-08-02

**Authors:** Shairul Izan, Danny Esselink, Richard G. F. Visser, Marinus J. M. Smulders, Theo Borm

**Affiliations:** ^1^Plant Breeding, Wageningen University and Research Wageningen, Netherlands; ^2^Department of Crop Science, Faculty of Agriculture, Universiti Putra Malaysia Serdang, Malaysia

**Keywords:** chloroplast genome, *de novo* assembly, *Solanum*, *Aegilops*, *Paphiopedilum*, DNA sequencing, whole genome shotgun sequencing, *k*-mer analysis

## Abstract

Whole Genome Shotgun (WGS) sequences of plant species often contain an abundance of reads that are derived from the chloroplast genome. Up to now these reads have generally been identified and assembled into chloroplast genomes based on homology to chloroplasts from related species. This re-sequencing approach may select against structural differences between the genomes especially in non-model species for which no close relatives have been sequenced before. The alternative approach is to *de novo* assemble the chloroplast genome from total genomic DNA sequences. In this study, we used *k*-mer frequency tables to identify and extract the chloroplast reads from the WGS reads and assemble these using a highly integrated and automated custom pipeline. Our strategy includes steps aimed at optimizing assemblies and filling gaps which are left due to coverage variation in the WGS dataset. We have successfully *de novo* assembled three complete chloroplast genomes from plant species with a range of nuclear genome sizes to demonstrate the universality of our approach: *Solanum lycopersicum* (0.9 Gb), *Aegilops tauschii* (4 Gb) and *Paphiopedilum henryanum* (25 Gb). We also highlight the need to optimize the choice of k and the amount of data used. This new and cost-effective method for *de novo* short read assembly will facilitate the study of complete chloroplast genomes with more accurate analyses and inferences, especially in non-model plant genomes.

## Introduction

Chloroplast genomes are frequently used in systematics and phylogeography because of the simplicity of the structure of its circular genome, its predominantly clonal inheritance along the maternal line, as well its high copy number in the cell (Palmer and Stein, 1986; Moore et al., 2006; Ma et al., 2013). The chloroplast genome is often perceived to have a low amount of sequence variation, and the use of the genome has therefore been mostly confined to studies at the interspecific and interfamilial levels (Jansen et al., 2007; Moore et al., 2007; Xi et al., 2012; Barrett et al., 2013). Recently comparative analyses of complete chloroplast sequences showed that the perception of low variation of chloroplasts within species is wrong when looking at the genomic scale (Whittall et al., 2010; Besnard et al., 2011; Kane et al., 2012). Kane et al. (2012) suggested that the whole chloroplast genome could be used as an ultra-barcode for identifying plant varieties. Furthermore, using one or few regions of the chloroplast genome is not the appropriate approach to describe the level of variability of the chloroplast genome. Therefore, using the complete chloroplast genome will undoubtedly be the best way to exploit the information in this organelle genome.

Chloroplast DNA can traditionally be obtained by a chloroplast enrichment strategy using a sucrose gradient (Moore et al., 2006) or high salt method (Bookjans et al., 1984). These strategies require large amounts of starting materials (∼5 g tissue), which may be challenging for endangered plant species or herbarium samples. Some plant groups may have a high content of polysaccharides, polyphenols, and/or terpenoids, which also poses a challenge to obtain high quality cpDNA (Vieira Ldo et al., 2014). Using PCR the complete chloroplast genome can be amplified in the form of a series of long, overlapping PCR fragments. This approach requires appropriate primer design as well as high quality DNA to ensure successful long range amplifications. The primers for these reactions have been designed on conserved gene sequences (Goremykin et al., 2003; Jansen et al., 2005), which work reasonably well across species. The implementation suffers from differences in gene organization among plant species (Atherton et al., 2010).

Next generation whole genome ‘shotgun’ (WGS) sequences of plant species often contain 5% or more reads that are derived from the chloroplast (Bakker et al., 2016). This offers an alternative way to obtain chloroplast genomes. The chloroplast reads are generally identified from the WGS reads and aligned into a chloroplast genome from a reference species. Such an alignment-based method has been a method of choice to do the sequence comparison during recent years. A comprehensive review about this method was Vinga et al. (2012). However, as structure and function in a genome may diverge over evolutionary time, such alignment-based methods may become unreliable for taxa for which no close relative exists with a high quality chloroplast genome. They may also become computationally unaffordable when dealing with very large datasets of sequences (Vinga et al., 2012 but see Bakker et al., 2016). Several alignment-free methods have been proposed to tackle those limitations and one of them is an approach based on *k*-mer frequency tables. The *k*-mer based approach may be the most developed alignment-free method (Chan and Ragan, 2013). A *k*-mer is an exact substring of DNA sequence of defined length (*k*), whose frequency in a set of DNA sequences can simply be counted (Marçais and Kingsford, 2011). Applying statistics on the sharing of *k*-mers between samples provides an estimate of genetic distance (Bonham-Carter et al., 2013). *K*-mer frequency tables are also used to distinguish sequencing errors from genuine sequences (Kelley et al., 2010) as sequencing errors are presumed to be random in nature thereby generating unique or low-frequency *k*-mers, while genuine sequences occur at a certain *k*-mer frequency, depending on the frequency of sequences in the target genome and the depth of sequencing in the WGS dataset. *K*-mer frequency tables have also been used to detect repeated sequences in the genomes (Kurtz et al., 2008), exploiting the fact that *k*-mers derived from a particular repeat of a certain copy number in the genome will have a similar frequency.

From the *k*-mer frequency tables, *k*-mer frequency distribution histograms can be derived (Chikhi and Medvedev, 2014) which show the volume of *k*-mers occurring at each frequency in the dataset. These are used as a basis for assemblies of, e.g., bacterial plasmids [plasmidSPAdes (Antipov et al., 2016) and Recycler (Rozov et al., 2015)] and may be used for plant mitochondrial and plastid genomes as well. If a particular, highly abundant (extrachromosomal) sequence occurs at a certain frequency in the dataset, this leads to a (broad) peak in this histogram. If another highly abundant sequence occurs at twice that frequency in the dataset, then there will be another peak in the histogram – at twice the frequency. Chloroplasts generally contain an Inverted Repeat (IR) region, and naturally *k*-mers obtained from reads in this IR region will occur at twice the frequency of *k*-mers obtained from Single Copy (SC) regions of the chloroplast, so we expect chloroplast-derived *k*-mers to be contained in two peaks in the histogram – the second at exactly twice the frequency of the first. In this study we have used *k*-mer frequency histograms to identify the two peaks corresponding to chloroplast-derived *k*-mers, and used their approximate frequencies to select the corresponding *k*-mers from the underlying *k*-mer frequency table. These *k*-mers were subsequently used to select reads containing them, which were then used in a first round of assembly. After the first round of assembly, subsequent rounds of assembly and refinement lead to an automated semi-finished assembly of a chloroplast genome.

This study demonstrates the feasibility of a procedure that employs a *k*-mer frequency histogram to extract the chloroplast sequences from whole genome sequencing data without the use of a reference genome prior to *de novo* assembly of shotgun sequences obtained with the Illumina platform. We used NGS data obtained from three species notably a solanaceous species, a grass species and an orchid species with a range of nuclear genome sizes (950 Mb–25 Gb) to demonstrate the universality of our approach. One of our cases is a novel chloroplast genome for an orchid species from the genus *Paphiopedilum*, which have a very large nuclear genome size (25–25 Gb).

## Materials and Methods

### Source of Sequencing Data Sets

Whole genome paired-end sequences of *Solanum lycopersicum* and *Aegilops tauschii* were downloaded from the sequence read archive of Genbank^1^. The WGS dataset for *Paphiopedilum henryanum* was generated for this study (**Table 1**) using fresh leaves of *P. henryanum* obtained from Hortus Botanicus in Leiden, the Netherlands. The DNA isolation was carried out by combining a DNA extraction using the protocol as described in Fulton et al. (1995) with a DNEasy Plant Mini Kit (Qiagen), using the kit’s DNA binding column to bind and clean-up DNA. A barcoded sequencing library was constructed by BGI, China, who also performed the 100 bp paired-end sequencing on an Illumina Hiseq2000 platform in a single lane along with 10 other samples from a separate experiment. For simplicity, from here onward we will refer to the analysis of WGS datasets obtained from *S. lycopersicum, Ae. tauschii* and *P. henryanum* as case studies 1, 2, and 3, respectively.

**Table 1:** Species used in the study and their SRA number.

	Haploid genome		NCBI SRA
Species (n)	size (bases)	Group	number
(1) *Solanum lycopersicum (2n)*	950 Mb	Dicot	SRR404081
(2) *Aegilops tauschii (2n)*	4–5 Gb	Monocot	SRR124187
(3) *Paphiopedilum henryanum (2n)*	25–35 Gb	Monocot	Own data

### Bioinformatic Analyses

#### Overview of the Approach

**FIGURE 1:**
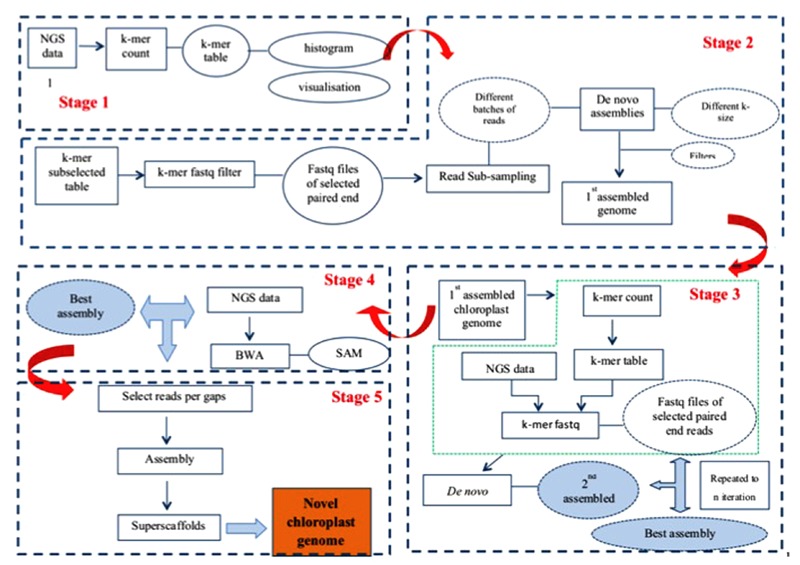
Workflow of our assembly pipeline.

Our assembly approach comprises five stages as illustrated in **Figure 1**. As the nuclear genome complement of different genomes results in differently shaped *k*-mer frequency distribution histograms, and as chloroplast DNA concentrations in WGS samples vary considerably, a visual inspection of *k*-mer frequency histograms is required between stages 1 and 2, where the user decides which *k*-mer frequency range to include in the analysis. While no human intervention is explicitly required between the other stages (2–5) of the pipeline, many optional parameters can be varied should the user require so, and the staging offers a convenient way for the user to monitor progress and output (assemblies) after each stage of the pipeline. Each stage is implemented as a separate PERL script, calling upon a large library of secondary PERL scripts, compiled C programs and external software (e.g., SOAPdenovo, BLAST) to perform its tasks. The pipeline software can be downloaded from http://secure.plantbreeding.nl/chloroplast/software/.

#### Data Preparation

Prior to stage 1 the user has to prepare the dataset by putting all sequence reads in fastq format files in a single directory. In order to allow the program to figure out which files contain matching paired-end reads and which files contain single end reads, the user has to adhere to a simple file naming convention.

#### Stage 1: Obtaining *K*-mer Frequency Tables and *K*-mer Frequency Histograms from WGS Datasets

The script implementing stage 1 produces alphabetically sorted *k*-mer tables with *k*-mer size 31 by default. In these *k*-mer tables, *k*-mers and their exact reverse complement are counted as a single ordinal *k*-mer. This ordinal *k*-mer is chosen from the two options in such a way that the middle nucleotide is always either ‘A’ or ‘C’ – if it isn’t then the *k*-mer is reverse complemented before being counted. After counting, a *k*-mer frequency histogram is produced from the tables. The *k*-mer frequency histograms are converted to histograms representative of the underlying data volume by multiplying the number of different *k*-mers occurring at each frequency with the frequency itself. We will refer to these histograms as *k*-mer volume histograms. To aid visualization, a series of binned histograms is produced with frequency bin sizes of 10, 25, 100, and 250.

#### Visual Inspection of *K*-mer Frequency Histograms

As each plant cell contains multiple chloroplasts, unless special precautions are taken during DNA sample preparation, molar concentration of chloroplast DNA in the WGS sample will be higher than that of nuclear DNA. Moreover, because chloroplasts most often contain an exactly duplicated Inverted Repeat (IR), the chloroplast DNA derived *k*-mers will produce a pair of peaks in the *k*-mer frequency histogram that can be easily distinguished from any other peaks because of their fundamental relation: The second (IR) peak occurs at twice the frequency of the first Single Copy (SC) region peak. The user then imports these *k*-mer frequency histograms into his/her favorite graphing package, and on the basis of the location of the peaks representing chloroplast sequence read derived *k*-mers decides where to set *k*-mer frequency boundaries.

#### Stage 2: Obtaining Chloroplast Specific Reads and Initial Assembly

The frequency boundaries set by the user are used in stage 2 to select, from the original *k*-mer frequency table, those *k*-mers occurring in this frequency range. These *k*-mers will, besides chloroplast derived *k*-mers, also contain *k*-mers derived from nuclear repeat-regions that coincidentally occur at the same frequencies. This *k*-mer table is then used to select, from the full WGS dataset, those reads that contain them. These selected reads are then sub-sampled into a series of batches of increasing size (by default starting at 100,000 read-pairs, with 100,000 read-pair increments, as the volume of data is known to affect the quality of the assembly), and automatically assembled using SOAP-denovo (v1.05) (Luo et al., 2012). SOAPdenovo is a De Bruijn graph-based assembler that can use a range of values for the *k*-mer size (*K*), and results have previously been found to be highly dependent on the value of *K* (Chikhi and Medvedev, 2014). Therefore we employed a range of different values for *K* (all odd values between 63 and 99). This yields a multitude of separate assemblies which are then filtered (by default using BLAST against the tobacco chloroplast genome as a representative of a good quality chloroplast genome) to remove any contig or scaffold that does not seem to be chloroplast-related (putatively repeats from the nuclear genome), and size-selected to remove any contig or scaffold smaller than twice the size of *K* (as used in the assembly). The resulting filtered assemblies are subsequently subjected to a sanity check where excessively short or excessively long assemblies are discarded. This filter is by default based on previously observed length ranges for SC and IR regions, and is user-configurable. The remaining assemblies are then ranked according to: (a) the number of scaffolds they consist of (fewer is better), (b) the number of gaps they contain (fewer is better) and (c) the total length of the assembly (longer is better). The best assembly is the one considered optimal for these three criteria at the same time, as to avoid assemblies that, e.g., satisfy assembly length at the expense of a low number of scaffolds. The best assembly is used in the next stage.

#### Stage 3: Iterative Refinement of Read Selection and Assembly

As discussed, the selection of *k*-mers in a set frequency range means that *k*-mers derived from nuclear genomic repeats coincidentally occurring at these frequencies are also selected. While enrichment of the dataset for chloroplast-derived reads is certainly achieved, the repeat region-derived reads co-selected because of this *k*-mer table contamination can be considered problematic. In the previous stage we tried to alleviate this by using BLAST and a size filter, but this carries the risk that some small fragments of genuine chloroplast sequence or highly deviant chloroplast sequences are lost. Stage 3 iteratively uses the putatively pure chloroplast derived assembly obtained in a previous iteration (or stage 2 for the first round) to select reads and re-assemble. To this end, a *k*-mer table is obtained from the chosen assembly, which is then used as described in the description of stage 2 to select reads, which are then assembled and filtered as described previously. Assemblies are ranked to produce a new best assembly until either no better assembly is produced or until a set limit on the number of iterations is reached. In addition to the assembly performed by SOAPdenovo, this stage employs its own assembly algorithm that looks for remaining overlap between scaffolds and contigs produced by SOAPdenovo, and where possible assembles these, taking into account the fact that a circular genome with an inverted repeat is expected (two aspects that existing assembly programs are unaware of). The final output of stage 3 is a new best assembly that is used in the next stage, and which may consist of linear or circular fragments. As the read-pair insert sizes attainable with current short read technology do generally not span a complete IR region, the exact relative orientation of the Short Single Copy (SSC) and Long Single Copy (LSC) regions cannot be determined. This assembly pipeline can (in case a circular assembly can be made) output either a set of three linear fragments (putatively representing LSC, IR, and SSC), two separate assemblies for both possible circular configurations, or just one (randomly chosen) circular assembly. Stages 4 and 5 require the last option, and it is left to the user to find the correct relative orientation of the LSC and SSC (to be validated for instance using long range PCR).

#### Stage 4: Scaffold Extension and Spanning-Read Based Re-scaffolding

The newly assembled genome resulting from stage 3 may or may not be circular, and if not circular it may or may not consist of multiple unconnected scaffolds, each of which may or may not contain gaps. The purpose of step 4 is to iteratively connect linear scaffolds remaining from stage 3 by extending and connecting scaffolds with additional sequence reads until scaffold ends overlap or by finding read-pairs spanning gaps between scaffolds. Stage 4 is skipped if stage 3 delivered a circular assembly. Briefly, all the raw reads are aligned back to the assembly using BWA and those (paired-end or single) reads that extended outside the gaps are picked. Each scaffold-end will produce a separate set of (paired-end) reads which are then assembled to obtain new scaffolds. These new scaffolds are added to the previous round best assembly and used as input to the internal sequence assembly algorithm and subsequently filtered as described under stage 3, producing a new assembly for use in the next iteration. Iterations are terminated if either (a) the resultant assembly is circular OR (b) the quality of the assembly does not improve (per the same criteria used to find the best assembly) OR (c) until a set limit on the number of iterations is reached. After the last iteration, if the resultant assembly is not circular already, raw reads are mapped back (BWA) against the resultant scaffolds and any read connecting scaffold-ends is selected and counted in a scaffold-end connectivity matrix. This scaffold-end connectivity matrix is combined with the scaffold sequences and used by the internal sequence assembly algorithm to produce a new assembly, placing N’s in gaps that are bridged by gap-spanning reads. Again, this may in some cases lead to construction of a circular assembly.

#### Stage 5: Gap Filling

After stage 4 gaps may remain in the sequence. These gaps are putatively caused by systematic (sequence dependent) low coverage in such regions, which should be considered an artifact of the Illumina sequencing technology used (Minoche et al., 2011). As we have used variable sized batches and various settings for *K* during the assembly, sufficient reads covering these low coverage areas may still remain unused in the dataset. Stage 5 attempts to fill the gaps by focussing only on reads covering such gaps, again assembling (using SOAPdenovo) variable sized batches of reads with a range of values for *K*. To this end, gap-context sequences (default 500 bp on either side of the gap) are extracted from the previous best assembly (either the previous iteration or stage 4), and used to produce a *k*-mer table for positive selection of reads. The regions of the previous stage best assembly scaffolds that are outside the defined gap-context are used to produce a second *k*-mer table that, after comparison with the positive selection *k*-mer table, is exported as a negative selection *k*-mer table. Raw reads are filtered using the positive selection *k*-mer table, retaining any read containing a *k*-mer from this set. Subsequently this subset is filtered using the negative selection *k*-mer table, discarding any read containing a *k*-mer from this set. The resulting set of reads is then assembled in variable sized [default 1000 read (-pair)s, with 1000 read (-pair)s increment] batches with SOAPdenovo using a range of values for *K* (odd values between 63 and 99). This delivers a number of scaffolds, which are then re-scaffolded using the internal assembly algorithm before being size filtered, discarding any scaffold shorter than *K* base-pair. The remaining scaffolds are then, one by one, combined with each separate gap context sequence using the internal sequence assembly algorithm, and ranked (for each of the gaps separately) to find the best gap-closing assembly. Finally, the best gap-closing assemblies (if any) are used to replace the gap context sequences in the original assembly, and the whole process repeats iteratively until either (a) all gaps are closed OR (b) until assemblies do no longer improve OR (c) a set limit on the number of iterations is reached.

## Results

### Determining Chloroplast-Derived *K*-mers Based on the *K*-mer Frequency Distribution

**FIGURE 2:**
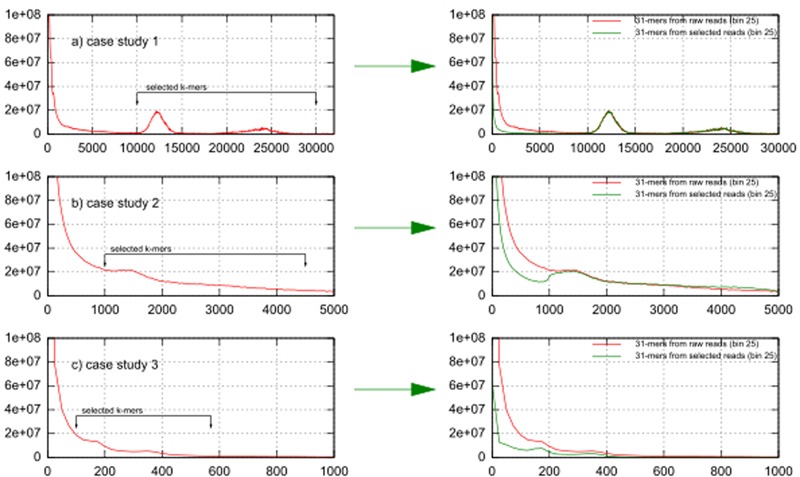
*K*-mer frequencies distribution of case studies 1, 2, and 3 before and after the *k*-mer selection. **(a)** In case study 1 (tomato) the nuclear haploid genome size is 950 Mbp, **(b)** in case study 2 (*Aegilops tauschii*) it is 4–5 Gbp, **(c)** in case study 3 (*Paphiopedilum henryanum*) it is 25–35 Gbp.

**Figures 2a–c** show *k*-mer volume histograms (binned per 25 frequencies) of the raw reads of case studies 1, 2, and 3. The two expected peaks for *k*-mers derived from the chloroplast genome sequences are clearly visible as sharp peaks in case study 1 (at 1200× and 2400× coverage), they were flatter in case 3 (at 170× and 350× coverage) (**Figures 2a,c**), while in case 2 only one peak (at 1500× coverage) could be discerned (**Figure 2b**). To see the effect of *k*-mer based read selection for chloroplast reads, we overlaid the *k*-mer volume histogram from the raw reads with the *k*-mer volume histogram of the reads picked out using the selected *k*-mers in the left part of **Figure 2**. In all datasets the volume of *k*-mers specific to erroneous sequences and to the nuclear genome were significantly reduced while the volume of *k*-mers in both chloroplast peaks essentially remained the same. This indicates that our selection enriches for chloroplast sequences.

### Extracting Chloroplast Reads and *De Novo* Assembly

Each case study contained between 15 million and 198 million raw read pairs. Following the *k*-mer based extraction of chloroplast reads from the raw reads of the case study, significant read reductions were seen across the stages. **Table 2** presents the total number of read-pairs in a dataset as well as the number of read-pairs used in stages two and three. Across three case studies a reduction by almost 40% of the number of read pairs is seen in stage two.

**Table 2:** Summary statistics before and after the fetching of the chloroplast reads.

	Case	Case	Case
	study 1	study 2	study 3
Genome size	950 MB	4–5 GB	25–35 GB
Total no of raw reads (pairs)	198 264 041	86 067 571	15 142 939
Total no of reads after stage 2 (pairs)	32 701 410	51 717 173	6 172 495
Total no of reads after stage 3 (pairs)	14 855 294	1 582 279	213 669

To investigate the optimum assembly for each case study, *de novo* assemblies with different batches of subsampled read pairs and *k*-sizes were performed. Basically, the pipeline gave a candidate best assembly at the end of stage 3 based on (1) the lowest number of scaffolds, (2) the fewest gaps and (3) the longest assembly length (within the allowed range). In case studies 1 and 2, inspection of the assembly statistics of all assemblies produced in stage 2 revealed that the automatically chosen assembly with the fewest number of scaffolds was either too long or contained an excessive number of gaps. Therefore, in these cases we manually selected an alternative best assembly based on minimal number of scaffolds plus gaps, with the longest length in the allowed range. In contrast, the automatically selected best assembly was a reasonable choice in case study 3 and thus did not need manual selection. In addition, we also investigated the efficacy of stages 4 and 5 for scaffold expansion or re-scaffolding and the gap filling. **Table 3** shows the statistics of the best assembly after stages 4 and 5. From our observation, all case studies showed that the stages 4 and 5 helped to merge scaffolds and fill the gaps. As example, in case study 3, eight scaffolds were merged and two gaps resolved in stages 4 and 5 compared to the underlying SOAPdenovo assembly (12 scaffolds with 3 gaps).

**Table 3:** Comparison of the SOAPdenovo assembly and *de novo* assembly derived after stages 4 and 5 from the proposed pipeline.

Case	Number of	Number	Total assembly	Total reference
study	scaffold	of gap	length	length
**Case study 1**				
SOAPdenovo	3	0	130 181^∗^	155 461^a^
Our approach	1	0	155 461	
**Case study 2**				
SOAPdenovo	9	4	114 806^∗^	135 685^b^
Our approach	2	2	135 760	
**Case study 3**				
SOAPdenovo	12	3	122 051^∗^	174 417^c^
Our approach	4	1	156 087	

*^∗^Contained only one copy of IR.*

*^a^*Solanum lycopersicum* chloroplast, complete genome (accession number NC007898.3).*

*^b^*Aegilops tauschii* cultivar AL8/78 chloroplast, complete genome (KJ 614412.1).*

*^c^*Cypripedium japonicum* chloroplast, complete genome (KJ 625630.1).*

### Mummer Analysis of Reference and *De Novo* Genomes

**FIGURE 3:**
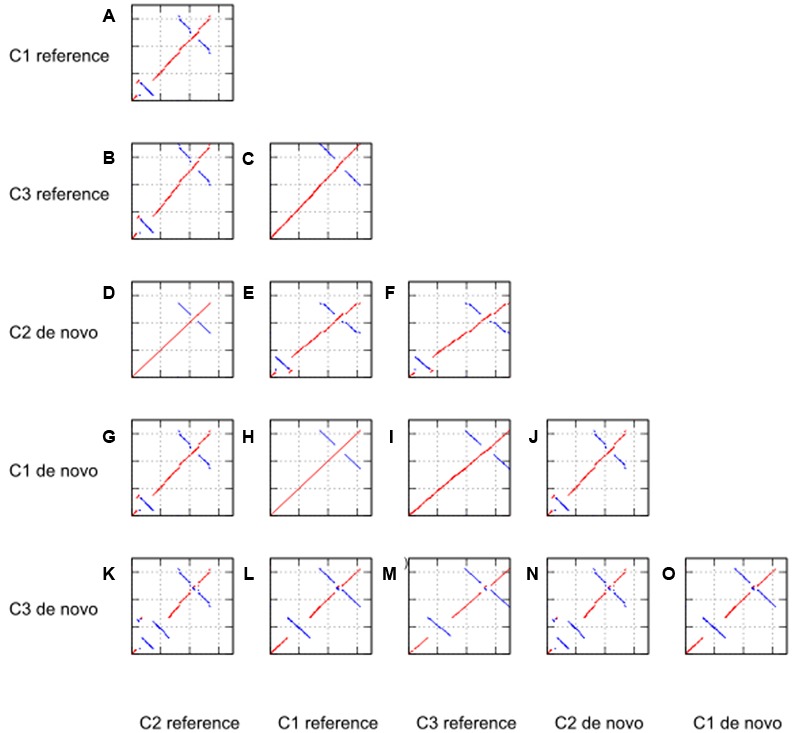
Dotplot analyses against reference genome and *de novo* assembled genome for case study 1 (C1, tomato), 2 (C2, *Ae. tauschii*), and 3 (C3, *P. henryanum*).

To detect any large structural variants such as inversions, insertions or deletions in the *de novo* assembled genomes, dot plot analyses were using MUMmer (Delcher et al., 2003). **Figure 3** displays the dotplots comparing all three *de novo* genomes as well as three reference genomes in all 15 possible combinations. Appropriate reference chloroplast genomes were downloaded from Genebank, NCBI with accession number NC007898.3, KJ614412.1 and KJ625630.1, respectively. As no reference genome is available for case study 3, we used a complete chloroplast genome from a related species.

From the dotplot analyses of only the reference genomes against each other (sub-figures a, b, and c), we note that the chloroplast of *Ae. tauschii* (KJ614412.1) has an inversion in the LSC region of about 13 860 bp length. The structure of the other two reference genomes was comparable without large structural variants. The inversion in the *Ae. tauschii* reference genome was also detected in our *de novo* assembly of case study 2 (as shown in sub-figure k). Moreover, we concluded the inversion in *Ae. tauschii* chloroplast genome was a genuine event as it was also supported by read mapping of the raw reads against the *de novo* assembled genome.

Interestingly, we also found two large structural changes in the *de novo* chloroplast assembly of case study 3 (sub-figure m). These structural variants in the *Paphiopedilum* species chloroplast genome are reported here for the first time. The first structural variation is an inversion in the LSC region. This inversion is absent in the reference genome of a related orchid species (*Cypripedium japonicum*). Secondly, we observed an IR expansion into the whole SSC region. Both these structural variations are absent in the other genomes including the orchid species *C. japonicum*. In addition, we conclude that all inversions are genuine events as they are supported by the read mapping (not shown).

### Mapping and *De Novo* Assembly of Sequence Reads

**FIGURE 4:**
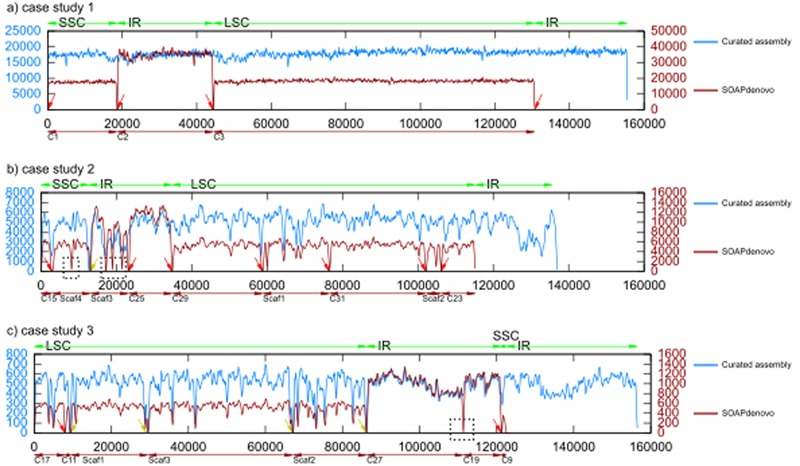
Comparison of read coverage between the assembly from SOAPdenovo and the curated assembly.

The raw reads were aligned against the *de novo* assembled genomes to verify the detected structural variation as well as to detect any miss-assemblies in the *de novo* assembled genomes. The read alignments were performed using BWA with default parameters. The mean coverage of the reads varied considerably among these three case studies (17822, 4396, and 497 times coverage for case studies 1, 2, and 3, respectively) illustrating that different DNA sequencing datasets contain different numbers of chloroplast reads. **Figure 4** shows comparison coverage plots of genomes assembled using our pipeline and unaltered assembly from the SOAPdenovo assembler. The assembly that SOAPdenovo produced only contained one copy of IR. The read coverage (*y*-axis) was plotted against the genome position and has been averaged using a window of 100 bp (*x*-axis).

In general, read coverage was sufficient to detect any miss-assemblies. Coverage plot comparison between the genome assemblies in each case study also demonstrated that our pipeline successfully assembled the scaffold across the low coverage regions. In contrast, SOAPdenovo assembler left gaps in the scaffolds (black boxes). This illustrated the power of the scaffold expansion, re-scaffolding and gap filling implemented in our pipeline leading to better quality of chloroplast genome assembly. Worth to mention, the zero coverage at the start and end of the genome (circular) of scaffolds (linear) characterized by red arrow was due to the pseudo-circularization – addition of a copy of the first N basepairs to the end of the assembly. This was done to facilitate the read mapping of the overhanging reads, which were used to connect two scaffolds. Beside the artifact because of pseudo-circularization, we also found, there were several positions pointed by the yellow arrow in the assembly of case studies 2 and 3 with zero read coverage, representing gaps in the genome assembly. This also suggests that the assembly will not improve anymore with this particular dataset.

### Variant Calling

Pairwise alignments for *de novo* assembled genome with their reference genome were conducted to call for variants. The result of variant calling is represents in **Table 4**. We do not present the pairwise alignment from case study 3 because we encounter a large number of variants across the genome, including two large structural variations. This large difference is due to the fact that the reference was from a related species and clearly the two species were too far diverged. We investigated the pairwise alignment from both other case studies and variants that were called included insertions or deletions (INDEL) and mismatches (SNP;s). Remarkably, we only found only one mismatch in the alignment of case study 1 at the position 127 404 bp which is located in the IR region. On the other hand, we successfully called 13 variants in the case study 2 consisting of 10 INDELs and three mismatches. Looking at those locations, we found five length variants of a homopolymer region.

**Table 4:** Variant calling for case studies 1 and 2.

			Position in the
Case study	Type	Variants	assembled genome
Case study 1	Mismatch	G (ref) > T (ass)	127404
Case study 2	Insertion	AGGTACCTAA	7653–7662
	Insertion	Homopolymer T region	18272–18274
	Insertion	Homopolymer A region	18614
	Insertion	Homopolymer A region	34160
	Insertion	CT	43329–43330
	Insertion	Homopolymer A region	56672–56673
	Mismatch	CTCTC (ref) > TCTCT (ass)	76298–76302
	Deletion	Homopolymer A region	78860
	Insertion	TTTACTTTTATGTTTTATTTG	107322–107342
	Insertion	GCAATAATCTACTAAAAAAA	109678–109697
	Mismatch	G (ref) > N (ass)	109894
	Mismatch	T (ref) > N (ass)	109893

## Discussion

### Chloroplast Genomes from Next Generation Sequencing Datasets

A chloroplast genome sequence provides information for addressing various biological questions, including phylogenetic analysis (Oxelman et al., 1997; Goremykin et al., 2003; Capella-Gutierrez et al., 2014). Furthermore, since the chloroplast genome is inherited uniparentally and is not subject to recombination during gametogenesis like the nuclear genome, it is an ideal locus for barcode analyses (Austerlitz et al., 2009; Hollingsworth et al., 2009; Li et al., 2015). The present study shows that it is possible to assemble high quality complete chloroplast genomes from whole genome shotgun (WGS) sequencing datasets using a largely automated pipeline. As next generation sequencing technology advances, more WGS data will become available to the researcher. Those data could be exploited using the approach outlined here in order to provide an easy and cost-effective way to construct complete chloroplast genomes. In this way it will be possible to reliably mine these resources for information on the chloroplast genome.

We also hope that our approach can help to increase the number of available chloroplast genomes. This will open up the possibility to do comparative analyses. In spite of the small size of the chloroplast genome, many fundamental characteristics such as functional sequences outside the coding sequences (promoter, terminator, replication origin), detection of selective signatures in gene sequences as well as mutational rates and their mechanism (Raubeson et al., 2007) are poorly described. Those hypotheses can be critically addressed by comparative studies.

### Comparison with Existing Pipelines

Our newly developed approach enables us to fetch chloroplast sequences from WGS sequencing reads without prior knowledge about the sequence and without additional effort during DNA isolation, and subsequently use those in a *de novo* assembly. This approach is different from existing protocols and tools to assemble chloroplast genomes, which require either a physical enrichment (e.g., specific isolation of chloroplast DNA) (Dong et al., 2013; McPherson et al., 2013; Vieira Ldo et al., 2014) or an *in silico* enrichment (alignment of WGS reads to a chloroplast reference) of the dataset for target sequences (Nock et al., 2011; Zhang et al., 2011; Kane et al., 2012). Our approach takes advantage of the known (LSC-IR-SSC-IR) chloroplast structure and the resulting, predicted, structure in the *k*-mer frequency distribution. In comparison to for instance the PlasmidSPAdes (Antipov et al., 2016) pipeline, selecting chloroplast reads based on the *k*-mer frequency distribution pattern of WGS instead of blastn (as used in PlasmidSPAdes) produced a better dataset with low coverage of reads from the nuclear genome of the plant, which reduced fragmentation and miss-assemblies in the *de novo* assembly process using SOAPdenovo. In addition, our pipeline illustrates the power of scaffold expansion, re-scaffolding and gap-filling as we implemented it, leading to better quality chloroplast genome assembly. Indeed, our approach detected structural rearrangements regardless of the availability or the quality of a reference genome. The strategy is not limited to Illumina data, but the current pipeline makes use of both pairs of the paired-end reads during the assembly, so it will need some adjustments when using long read technologies. Furthermore, the approach is indifferent to the ploidy level of the species or the level of heterozygosity as we assemble a chloroplast genome, not a nuclear haplotype.

Our approach employs some publicly available software in combination with custom-made scripts, which are available on request. The merits of the strategy we followed is discussed below in general terms, as it may also be implemented using other software. For instance, there are many alternatives for the script producing *k*-mer tables. One may use GenomeTester4 (Kaplinski et al., 2015) or any of the tools reviewed and compared by Pérez et al. (2016), including Jellyfish (Marçais and Kingsford, 2011) and Tallymer (Kurtz et al., 2008).

### *K*-mer Frequency Distribution, Sequencing Error, Coverage Bias, and Genome Size

The distribution of *k*-mer frequencies in a whole genome DNA sequence dataset includes information on the underlying genomes as well as on characteristics of the sequencing run. As there is a large inverted repeat in the chloroplast, a bimodal *k*-mer frequency distribution is expected, with one peak (representing the inverted repeat) occurring at exactly twice the frequency of the other peak. This allows identification of these peaks in a *k*-mer frequency distribution. However, as there are other (e.g., genomic) sequences present in the dataset, there may be a significant background present of *k*-mers derived from these other sequences at similar frequencies as the choloroplast derived *k*-mers, and the amount of background is clearly influenced by the nuclear genome size, as can be observed in our three case studies. Several studies investigating the link between *k*-mer frequency distribution and sequencing errors have been carried out (Kurtz et al., 2008; Kelley et al., 2010; Liu et al., 2013). Random sequencing errors will generate a high peak with low coverage, and as the rate of sequencing errors increases, this “error-peak” on the left side of the frequency plot will increase in size, while other peaks will become smaller and also decrease in frequency, thus move to the left. Of course, if there are highly repetitive regions in the genome, with correspondingly higher *k*-mer frequencies, errors in the sequences generated from these repetitive regions will also occur at a larger rate, consequently giving rise to a widening of the error-peak. For large, complex, genomes it is expensive to generate sufficient coverage of the nuclear genome to be able to easily separate the peak corresponding with genomic DNA (“nuclear genome peak”) in the *k*-mer frequency histogram from the error-peak, and as a consequence, the “nuclear genome peak” may overlap the “error peak” and become an inseparable, very wide combined peak, even overlapping the “chloroplast peaks,” as can be seen in case study 3, and to a lesser degree in case study 2. On the other hand, for case study 1 the “nuclear genome-peak” is well separated from both the “error peak” and the “chloroplast peak.” Case study 1 is an excellent example of the desired separation of the sequencing error, while the datasets of case studies 2 and 3 might benefit from more sequencing data – better separation between the desired “chloroplast peaks” and the undesired “error peak” and “nuclear genome peak” would improve the selectivity of the *k*-mer frequency based filtering of reads. As was intended, we noticed in all cases that the coverage of *k*-mers specific to error and nuclear genome were reduced significantly after the *k*-mer selection while the coverage of peaks belong to chloroplast sequences remained the same or slightly reduced as seen in case study 3.

Wherever frequencies of *k*-mers obtained from the nuclear genome overlap the “chloroplast peaks,” reads derived from the corresponding, evidently repetitive regions, from the nuclear genome will also be selected and included in the assembly process. The effect that this might have on the chloroplast assembly depends on several factors. First of all it depends on the lengths of the repeating units – if these are small (e.g., <500 bp), the resulting assemblies will be also be small, and may be removed on the basis of their size alone. If the repeating units are large (e.g., >10 K) and high frequency, then this would be a novelty and mean that a large proportion of the nuclear genome would be contained in such repeats. Such long repeats are also very easy to remove as long as they don’t bear any resemblance to known chloroplast genomes. Insertions of parts of a chloroplast genome into the nuclear genome might be an interesting problem if these insertions would happen be large and would happen within repetitive regions – in such cases chimeric scaffolds may be expected. Outside the repetitive regions the non-repetitive nuclear genome will give rise to relative low frequency *k*-mers, which would therefore not be selected, and which would therefore not lead to inclusion of larger regions of nuclear genome derived reads into the assembly process. While this may, depending on overall sequence coverage, lead to some confusion in the assembler, this should not lead to many problems in the downstream analysis. Incidental insertion of parts of a chloroplast genome into the nuclear genome should also not lead to detection of SNP’s in the chloroplast – the SNPs will give rise to *k*-mers occurring at frequencies corresponding to the nuclear genome, and the underlying reads will either not be selected on the basis of their *k*-mer frequencies or, if they happen to be selected, add little coverage in the assembly process, and be consequently treated as sequencing errors and be removed.

The relative positions in the *k*-mer frequency histograms of the peaks corresponding to the nuclear genome and the chloroplast, in combination with their respective genome sizes can give us some insights into the number of chloroplast genomes per cell. From the perspective of chloroplast genome assembly, a fixed ratio between the number of nuclear genomes (Palmer and Stein, 1986) and chloroplast genomes is a worst case scenario: in WGS datasets of larger genomes the percentage of chloroplast derived reads would then be lower, necessitating disproportionally more sequencing in larger genomes to obtain a usable coverage of the chloroplast genome. In some cases it may even be appropriate to combine our method with a chloroplast DNA enrichment strategy.

Our data seem to indicate that the percentage chloroplast reads in a WGS dataset is not constant, but decreases when the nuclear genome size increases. This could be expected if the number of chloroplasts per cell is more or less constant, or regulated between tissues in the same way regardless of nuclear genome size, but it was not what Bakker et al. (2016) observed. This may be related to the fact that they only tested a limited range of genome sizes. On the other hand, the anecdotic case studies that we present here may be the ones deviating from the general trend.

### *K*-mer Size and Assemblies

The SOAPdenovo assembler is based on a De Bruijn-based graph which breaks the reads into *k*-mers of defined size before assembling them into contigs (Pevzner et al., 2001). After initial *k*-mer based graph construction, several steps refer back to the original underlying data to resolve some of the issues caused by the short length of *K* – most notably resolution of knots caused by repeat units smaller than the length of the reads yet larger than *K*. SOAPdenovo performed better for the chloroplast assembly than some other assemblers we tested (not shown). The robustness of the SOAPdenovo assembler relies on several competing effects that are difficult to quantify.

One important parameter is the *k*-mer size *K*. For instance, *K* smaller than some repeat sequences may cause tangling up in the De Bruijn graph, which, if very complex and unresolvable with the raw-read-data, may lead to contigs being broken up. Thus, we need large *K*. However, larger *K* will reduce the number of *k*-mers that can be extracted from a given sequence read – and as a consequence lead to fewer *k*-mers being extracted from a dataset overall and hence lowering of *k*-mer frequencies. Lower *k*-mer frequencies may make it difficult to distinguish good sequence from sequencing errors, and may eventually lead to problems in De Bruijn graph construction. Also, assuming random distribution of sequencing errors, the probability of a longer *k*-mer containing a sequencing error is larger, which will lead to more *k*-mers being included in the error-peak. Another effect is that if two contigs overlap by less than *k*-1 characters, this will create a coverage gap resulting in the break-up of a contig (Chikhi and Medvedev, 2014).

Another factor influencing the assembly process is the amount of data being used. More data does not necessarily improve assembly quality. Especially for extreme coverage data, and for non-random sequencing errors, assembly of larger datasets may give rise to alternative assemblies, one with the “proper” sequence, and one containing an “SNP.” Having alternatives for regions is not easily representable in FASTA format assembly output, and in SOAPdenovo it generally leads to fragmentation.

In the algorithm of the pipeline presented here we employed a range of different values for *K* in order to minimize the trade-off effects. We also employed a range of dataset sizes by including different numbers (“batches”) of reads in the assembly process. This yields a multitude of separate assemblies which are then filtered out using some filters. The remaining assemblies are then ranked accordingly and putatively best assembly was selected automatically. As seen in case studies 1 and 2 the automatic selection of a best assembly based on maximum assembly length and minimal (number of scaffolds plus gaps) may be more appropriate than maximum assembly length and minimal number of scaffolds alone. In contrast, in case study 3 the automatic selection of a best assembly based on maximum assembly length and minimal (number of scaffolds and gaps) was sufficient. This indicates that intelligent inspection of intermediary results for every stage in the pipeline is useful.

### Assemblies and Sequencing Bias

Compared to other studies that use reference sequences to extract chloroplast reads, the approach proposed here extracts the reads derived from the chloroplast solely based on the fact that they occur at the certain frequency in the *k*-mer frequency distribution of WGS data. By utilizing such an approach, we obtained reasonably high coverage of chloroplast genome across the case studies. Nevertheless, there are several gaps in the *de novo* assembled genome compared to the reference genome in case studies 2 and 3. Those gaps in the assembled genome may be caused by sequencing bias in the sequencing library. For instance, bias in the pre-sequencing amplification step could result in poor or no sequencing coverage in certain regions of the genome. Generally, a CG content sequencing bias has been observed. In accordance with our results, several studies, e.g., Dohm et al. (2008), Li et al. (2010), and Minoche et al. (2011) claim that even though there is sufficient average depth of sequence coverage within sequencing datasets, sequencing bias leads to region of no sequence coverage within sequencing datasets, resulting in multiple gaps in the assemblies, and hence a larger number of contigs and scaffolds even in small sized genomes such as bacteria and the chloroplast genome.

### Structural Differences, INDEL Detection and Homopolymers Length Polymorphism

The selected reads were assembled *de novo* instead of taking an alignment or reference guided *de novo* assembly approach. A *de novo* assembly offers additional possibilities for detecting structural differences that may be missed in other approaches. Moreover, our pipeline uses read coverage information, which provides for detection of sequence variation. We detected several structural differences in two out of three case studies. Even considering the general conservation of chloroplast genome, several structural differences were reported for nine grass species (Golenberg et al., 1993), Korean ginseng (Kim and Lee, 2004), and Pinus (Parks et al., 2009). Hence, it may be inappropriate to assemble the chloroplast genome for non-model species by alignment to a reference sequence of a related species because it may miss important structural differences but also because reads from repeated or homologous regions can generally not be distinguished in a mapping based approach – which may lead to identification of false SNPs in such regions. Another issue to be aware of is that half of variants detected in case study 2 were homopolymer length polymorphisms. This may be due to the fact that the reference genome of *Ae. tauschii* (KJ614412.1) was sequenced on the SOLiD platform while WGS dataset of case study 2 was sequenced by Illumina. It is known that Illumina sequencing is less affected by homopolymer length variation (Harismendy et al., 2009). It is also a known issue that SOLiD shows low coverage of AT-rich regions, while Illumina sequencing has been observed to have more problems with CG-rich regions (Morozova and Marra, 2008; Harismendy et al., 2009).

### Possible Enhancements

As is, the pipeline is configured to use a fairly naive in-house developed *k*-mer counting tool as part of the pipeline. Replacement by one of the available more performant alternatives [e.g., GenomeTester4 (Kaplinski et al., 2015) or any of the tools reviewed and compared by Pérez et al. (2016), including Jellyfish (Marçais and Kingsford, 2011) and Tallymer (Kurtz et al., 2008)] is straightforward – requiring the user to provide a simple wrapper script that takes FastQ files as input and produces a sorted *k*-mer table as output, and pointing the pipeline to this wrapper script. While replacement of the provided FastQ *k*-mer counting tool would undoubtedly result in immediate performance gains, it would also lead to additional external dependencies for the software, without changing the outcome, as *k*-mer counting tools essentially produce exactly the same result from the same input.

An assembler is vastly more complex than a *k*-mer counting tool, with many design choices that may affect the outcome. Replacement of SOAPdenovo by any other assembler is possible, and can be as easy as providing a simple wrapper script if there is a simple relationship between the data structures, configuration files, parameters and output of the alternative assembler and those of SOAPdenovo. If one considers that the parameter sweeps for K and dataset size that we do for SOAPdenovo may not be appropriate optimizations for other assemblers, implementing proper support for a different assembler may be more work (replacing the parameter sweeps), and require careful validation of results. Given that SOAPdenovo, in combination with our pipeline, produces adequate chloroplast assemblies we have not felt the need to implement different assembler options yet, however, we cannot rule out that other assemblers with other optimizations may produce better assemblies on datasets that are more challenging to SOAPdenovo.

Many *k*-mer counting tools and assemblers currently support multi-processing. From the perspective of our pipeline, this *k*-mer counting and assembly component multiprocessing is irrelevant as it is completely implemented at the component level and opaque to the pipeline. Given that the pipeline itself performs a multitude of similar tasks (n.b. parameter sweeps), it would be possible to make the pipeline itself multi-processing. While performance benefits are immediately evident, this would be a complex undertaking – requiring extensive changes and a framework to control resource (CPU, memory, disk) usage of components and maintain synchronization between sub-tasks.

Overall, there is potential for (particularly computational performance-related) improvements, however, for our purposes – generating finished or nearly finished chloroplast assemblies from WGS data with little user interaction – the current pipeline is adequate.

## Conclusion

The chloroplast genome certainly is a great resource of molecular markers in many studies including parentage analysis, hybridization, population and genetic structure and phylogeography. The pipeline described here provides a tool to extract chloroplast sequences from WGS sequences of plant species. Our newly developed pipeline was able to efficiently assemble the chloroplast genome across a range of nuclear genome sizes, and using it we discovered several structural rearrangements compared to published reference chloroplast genomes. This cost-effective approach will be particularly useful for exploring in the increasing number of WGS sequences from non-model species. In principle, our pipeline in combination with high throughput short read sequencing can greatly expand the scope of comparative genomics of the chloroplast genome in plants.

## Author Contributions

Conception of the study: SI, MS, and TB. Collection of material: SI. Production of the data: SI, DE, and TB. Software: TB. Data analysis: SI, MS, and TB. Writing of the manuscript: SI, RV, MS, and TB. All authors read and approved the final version of the manuscript.

## Conflict of Interest Statement

The authors declare that the research was conducted in the absence of any commercial or financial relationships that could be construed as a potential conflict of interest.

## Footnotes

1 http://www.ncbi.nlm.nih.gov/sra
